# An Evaluation of a SVA Retrotransposon in the *FUS* Promoter as a Transcriptional Regulator and Its Association to ALS

**DOI:** 10.1371/journal.pone.0090833

**Published:** 2014-03-07

**Authors:** Abigail L. Savage, Thomas P. Wilm, Kejhal Khursheed, Aleksey Shatunov, Karen E. Morrison, Pamela J. Shaw, Christopher E. Shaw, Bradley Smith, Gerome Breen, Ammar Al-Chalabi, Diana Moss, Vivien J. Bubb, John P. Quinn

**Affiliations:** 1 Department of Molecular and Clinical Pharmacology, Institute of Translational Medicine, The University of Liverpool, Liverpool, United Kingdom; 2 Clinical Neuroscience, Institute of Psychiatry, King's College London, London, United Kingdom; 3 School of Clinical and Experimental Medicine, College of Medicine and Dentistry, University of Birmingham, Birmingham, United Kingdom; and Neurosciences Division, University Hospital Birmingham NHS Foundation Trust, Birmingham, West Midlands, United Kingdom; 4 Academic Unit of Neurology, Department of Neuroscience, Sheffield Institute for Translational Research, University of Sheffield, Sheffield, South Yorkshire, United Kingdom; 5 MRC Social Genetic and Developmental Psychiatry Research Centre, Institute of Psychiatry, King's College London, London, United Kingdom; National Institute for Health Research Biomedical Research, Centre for Mental Health, South London, United Kingdom; and Maudsley NHS Foundation Trust and Institute of Psychiatry, King's College London, London, United Kingdom; 6 Department of Cellular and Molecular Physiology, Institute of Translational Medicine, The University of Liverpool, Liverpool, United Kingdom; Boston University School of Medicine, United States of America

## Abstract

Genetic mutations of *FUS* have been linked to many diseases including Amyotrophic Lateral Sclerosis (ALS) and Frontotemporal Lobar Degeneration. A primate specific and polymorphic retrotransposon of the SINE-VNTR-Alu (SVA) family is present upstream of the *FUS* gene. Here we have demonstrated that this retrotransposon can act as a classical transcriptional regulatory domain in the context of a reporter gene construct both *in vitro* in the human SK-N-AS neuroblastoma cell line and *in vivo* in a chick embryo model. We have also demonstrated that the SVA is composed of multiple distinct regulatory domains, one of which is a variable number tandem repeat (VNTR). The ability of the SVA and its component parts to direct reporter gene expression supported a hypothesis that this region could direct differential *FUS* expression *in vivo*. The SVA may therefore contribute to the modulation of *FUS* expression exhibited in and associated with neurological disorders including ALS where *FUS* regulation may be an important parameter in progression of the disease. As VNTRs are often clinical associates for disease progression we determined the extent of polymorphism within the SVA. In total 2 variants of the SVA were identified based within a central VNTR. Preliminary analysis addressed the association of these SVA variants within a small sporadic ALS cohort but did not reach statistical significance, although we did not include other parameters such as SNPs within the SVA or an environmental factor in this analysis. The latter may be particularly important as the transcriptional and epigenetic properties of the SVA are likely to be directed by the environment of the cell.

## Introduction

Genetic variation which alters the primary sequence of a protein has allowed tremendous insight into underlying mechanisms associated with predisposition, progression and severity of diseases. However, most genetic variation identified in candidate gene and genome wide association studies associated with disease processes is within non coding regions. This has led to a greater analysis and emphasis on the importance of gene-environment interactions in which tissue specific or stimulus inducible challenges target transcriptional regulatory domains to alter mRNA abundance underlying the disease process. Amyotrophic Lateral Sclerosis (ALS) is one disease in which such a mechanism may play a significant role, because although about 5% of ALS is familial (FALS), in most cases of ALS the patient has no family history of the disease (sporadic ALS; SALS). Nevertheless, cases with a significant genetic component can give us insight into which signal transduction pathways may be compromised in the development of the disease as they can highlight processes which may be targets for the challenges which trigger ALS.

*FUS* (Fused in sarcoma) found on chromosome 16p11.2 is a RNA binding protein. Mutations in its coding exons have been identified in some cases of FALS and it is therefore a candidate for genetic association with ALS [1]. The number of ALS cases attributed to mutations in the *FUS* gene is small; *FUS* mutations are present but rare in SALS at around 1% [2]–[5] and found in only 3–5% of FALS [6], [7]. Although rare genetic mutations in the *FUS* gene account only for a small proportion of apparently non-familial SALS, *FUS* positive inclusions have been found in the anterior horn of the spinal cord in SALS patients without *FUS* mutations, and in non-SOD1 FALS [8]. Whilst *FUS* is ubiquitously expressed, the levels of *FUS* may be critical for cell viability, and modulation of expression may be associated with the initiation or progression of ALS suggesting a role for the environment in modulation of levels of *FUS* gene expression. A differential response in gene expression to the stimulus could be modulated by the genotype thus allowing for a Gene x Environment interaction (GxE) in the initiation or progression of conditions such as ALS in which *FUS* is implicated. This would be consistent with a recent mouse model in which over expression of wildtype *FUS* caused progressive motor neuron degeneration in an age- and dose-dependent fashion [9]. We therefore undertook an analysis of the *FUS* locus to determine potential regions of genomic variation that are candidate domains to direct differential gene expression in response to environmental challenge.

Although it is difficult to accurately predict the regulatory domains for a particular gene other than the proximal promoter (often 0.5 to 1 kb upstream of the transcriptional start site), our group and others have demonstrated important domains for gene regulation can reside in both the most evolutionary conserved regions (ECRs) which are non-coding [10]–[12] and the highly polymorphic and often rapidly evolving variable number tandem repeats (VNTRs) [13]–[19]. In both cases the ECR or VNTR can be tens of thousands of bases from the major transcriptional start of a gene [20]. Genetic variants in both classes of domains are often clinical correlates of disease progression [10], [14], [21]. The searches for potential areas involved in transcriptional regulation can be aided by utilisation of ENCODE (encyclopaedia of DNA elements) data searching for the presence of potential transcription factor binding sites, active histones or DNase 1 hypersensitivity clusters [22], [23]. We performed such a bioinformatic analysis of the *FUS* locus and highlighted one large VNTR region 5′ of the *FUS* gene which overlapped active histones and other ENCODE data suggesting it might act as a transcriptional regulatory domain (Figure S1). Further analysis demonstrated the VNTR was part of a larger primate specific retrotransposon termed a SINE-VNTR-Alu (SVA) element. SVAs are the most recent family of retrotransposons to insert into the human genome with 2676 SVAs identified in the Hg19 release from UCSC genome browser [24]. There is considerable interest, but limited data available describing the role of retrotransposon elements in human health with 96 disease causing insertions having been identified as of 2012 [25]. In the ageing brain somatic retrotransposition has been demonstrated and this plasticity in the genome has been suggested to play a role in the diseases associated with an ageing population [26], [27]. Furthermore in tumours it has been shown that epigenetic modulation of retrotransposons in general including SVAs can vary in cancer progression, specifically, alterations in methylation patterns have been detected [28]. The SINE region of the SVA derived from the human endogenous retrovirus K10 (HERV-K10) has been used to classify SVAs into subtypes A-F with the age of each subtype ranging from an estimated 13.6 million years for the oldest (SVA A) to 3.2 million years for the youngest (SVA F) [29]. An additional subtype was identified that contains sequence from exon 1 of the MAST2 gene and associated CpG island at the 5′ end of the SVA and was named CpG-SVA, MAST2 SVA or SVA F1 [30]–[32]. The SVA in the *FUS* gene is classified as subtype D, or SVA D. Based on data from the UCSC browser using the human genome sequence release 19 as the reference genome (http://genome.ucsc.edu/), this particular element is found only in humans and chimpanzees amongst the primates.

Retroviruses, exogenous and endogenous, have been linked with ALS [33]. An increased prevalence of reverse transcriptase (RT), a key enzyme in the retrovirus life cycle converting RNA to DNA, has been observed in the serum of patients with SALS [34], [35]. In the second study [34] the elevated RT enzyme levels were interpreted as indicative of involvement of an endogenous retrovirus rather than an exogenous retrovirus as blood relatives also had elevated levels whereas spouses were the same as controls. A further study has implicated retrotransposons as having a role in ALS because HERV-K transcripts and RT protein were detected in autopsy brain tissue of patients with ALS along with the aberrant expression of TDP-43 [36]. These authors suggested targeting of activated genome-encoded retroviral elements may open new prospects for the treatment of ALS. The cellular environment that led to this increased expression of HERV-K transcripts and RT may be a global change that could influence the expression or activity of other retrotransposons in the genome for example epigenetic changes across mulitple loci of retrotransposons have been shown in cancer [28]. We hypothesised that the SVA upstream of the *FUS* gene could be one such domain. The activation does not have to lead to retrotransposition for it to affect gene expression in adjacent genomic loci, as alteration of epigenetic factors may modulate any transcriptional properties embedded within the SVA. Our hypothesis is that the SVA domain could have significant potential to modulate gene expression at the *FUS* locus and that the variation in the VNTR could support differential gene expression based on the challenge that the cell receives. Therefore we addressed the ability of the SVA D 5′ of the *FUS* gene to act as a classical transcriptional regulator in reporter gene constructs *in vitro* and *in vivo*. We further addressed its potential polymorphic variation and whether such variation acts as a predisposing factor for ALS.

## Materials and Methods

### Cell culture

The human neuroblastoma cell line SK-N-AS (American Type Culture Collection Resource Centre stock number CRL-2137) was maintained in Dulbecco's Modified Eagle's Medium (Sigma, D5672), 10% foetal bovine serum (ThermoScientific/Hyclone,), 1% penicillin/streptomycin (100 U/ml, 100 μg/ml; Sigma P0781), 1%(v/v) Non-Essential Amino Acids (Sigma, M7145) and 1%(v/v) 200 mM L-glutamine (Sigma, D7513), in 5% CO_2_ at 37°C.

### Generation of reporter gene constructs for use *in vitro*

All regions were amplified by high fidelity PCR from pooled mixed gender human genomic DNA preparations (G3041 Promega, USA) using *Pfu* DNA polymerase (Promega, USA). Primers used incorporated restriction sites for directional cloning within an added octameric linker sequence (underlined below, forward: *Nhe*I, reverse: *Bgl*II) and the first two PCR-cycles were performed at annealing temperatures matching template-specific sequences exclusively. The following primer sets were used: SVA (1240/1190bps, long/short alleles), (forward) 5′-GGCTAGCCGTGACTATTGCATACCTTGCCCCAGGCC-3′, (reverse) 5′- GAGATCTCGGAGAGGTTGTCATGGTACACAGACTGG-3′; TR/VNTR (862/812 bp, long/short alleles), (forward) 5′-GGCTAGCCCAGTTTTCCCTCAGACCCAGC-3′, (reverse) 5′-GAGATCTCGTTGGGGGTAAGGTCACAGATCAACAGG-3′. Amplified fragments were cloned into the firefly luciferase reporter gene expressing vector pGL3P, containing a SV40 minimal promoter element (Promega, USA). Correct cloning and sequence were verified by bi-directional sequencing using standardised primers.

Endogenous *FUS* expression in SK-N-AS cell line was confirmed by semi-quantitative RT-PCR from purified total RNA preparations using the following primers: (forward) 5′- AGGTGACTGTTTAGTGGGTAGGTC-3′ and (reverse) 5′-ATAGCCGGACACAGTATCTCACAC-3′.

### Cell transfection and dual luciferase assay

SK-N-AS cells were co-transfected with test constructs (firefly luciferase reporter gene) and an internal control construct, pMLuc-2 (*renilla* luciferase reporter gene; Novagen, USA) using TurboFect Transfection Reagent (ThermoScientific/ Fermentas, R0531) according to manufacturer's protocol in 24-well plate format. Transfectant was removed after 4 hours of incubation and exchanged with fresh medium and subsequent luciferase activity assays performed after 48 hours of incubation.

Luciferase activity of reporter constructs was measured using a Dual Luciferase Reporter Assay System (Promega, USA) using lysates from transfected cultured cells according to manufacturer's instructions. Assays were carried out on a Glomax 96-well microplate Luminometer (Promega, USA) using 20 µl of cell lysate. Measurements were averaged from 6-fold replicates to minimize pipetting errors and repeated at least three times to confirm results. Statistical analyses were performed using MSExcel software and a one tailed t-test to measure the significance of fold activity of the *FUS* SVA and TR/VNTR over the minimal promoter of the pGL3P vector *P<0.05, ***P<0.001, and to compare the activity of the alleles of the SVA and the TR/VNTR to each other # P<0.05.

### Construction of plasmids for *in vivo* fluorescent models

#### Generation of tomato reporter plasmid

Tomato gene sequence was PCR amplified from pG-tdTomato (a kind gift from Marco Marcello, University of Liverpool) using primers Tomato UP 5′- ATAGGAATTCCGTGTACGGTGGGAGGTCTA-3′ and Tomato DOWN 5′- GGCCGTCGACATCATTTTACGTTTCTCGTTC-3′ which introduce *Eco RI* and *Sal I* restriction sites, upstream and downstream respectively, for directional cloning into the plasmid pIRESGFP (kind gift from John Gilthorpe). The pIRES-GFP cassette was removed using *EcoRI* and *XhoI* restriction sites and replaced by the Tomato reporter gene, such that it was downstream of the chick β-actin promoter.

#### Generation of human *FUS* L-SVA and L- TR/VNTR *in vivo* reporter plasmids

The generation of the proximal *FUS* promoter reporter plasmid is described elsewhere (Kursheed et al. in preparation). Briefly, human *FUS* promoter sequences (−160/+84) were cloned into the Sac*I/*Bam*HI* sites of the promoter-less reporter vector phrGFP (Stratagene,UK) upstream of the GFP reporter gene. Identity was confirmed by sequencing and plasmid named ppGFP. The *FUS* SVA and isolated TR/VNTR sequences, both isotype ‘long’ allele were amplified by PCR from L-SVA and L-TR/VNTR reporter plasmids described above using standard Phusion polymerase conditions (NEB Biolabs) with the addition of 3% DMSO (v/v). The primers used are outlined below and included *NsiI* and *XbaI* restriction enzyme sites (underlined) to facilitate directional cloning: SVA UP 5′-TTGCATGCATGTGACTATTGCATACCTTGC-3′and SVA DN 5′-GACGTCTAGAGGAGAGGTTGTCATGGTACA-3′ and TR/VNTR UP 5′-TTGCATGCATCAGTTTTCCCTCAGACCCAG-3′and TR/VNTR DN5′-GACGTCTAGAGTTGGGGGTAAGGTCACAGA-3′. The resulting products were cloned into the *Nsil/Xbal* sites of *FUS* ppGFP and sequences were verified, this created L-SVA ppGFP and L-TR/VNTR ppGFP.

### Manipulation of chick embryos

Fertile chick eggs were incubated at 37.8°C for two days until they were approximately developmental stage 14 HH. 2–3 ml of albumen was removed and a window was cut in the egg. Embryos were staged according to Hamburger and Hamilton [37]. In those at stage 11–14 the vitelline membrane was removed to aid manipulation of the embryo. The lumen of the neural tube was injected with a solution containing 2–5 µg/µl of test DNA reporter plasmid, 1 µg/µl of Tomato plasmid (control for successful injection) in PBS containing 1 mM MgCl_2_ and 0.2%(v/v) fast green (to help visualisation). Injections were undertaken with a pulled micropipette made from a borosilicate capillary (Warner Instruments). Post-injection, DNA was immediately electroporated into the cells of the neural tube; gold plated electrodes of 3 mm length (Harvard Apparatus) were placed either side of the embryo with an internal gap of 5 mm and 5×50 ms square wave pulses with 100 ms gaps were delivered. Electroporated embryos were incubated at 37.8°C for 48 hours until they were approximately developmental stage E5 and then assessed for expression of plasmid DNAs. Electroporated embryos were dissected out and photographed using epifluorescent microscopy.

### Genotyping the VNTR of the *FUS* SVA

The following primers; forward 5′CAGTTTTCCCTCAGACCCAGCAC 3′ and reverse 5′GAGCTGTTGGGTACACCTCCCAGAC 3′ were used to amplify the TR/VNTR sequences within the SVA 5′of the *FUS* gene in a SALS and matched controls cohort from the King's College London MND DNA Bank by PCR. All participants gave ethically approved written consent to participate in the study, which was approved by the South London and Maudsley Ethics Committee (reference 222/02). The templates were 5 ng of genomic DNA from the SALS patient samples and matched controls and amplification reactions used Taq polymerase with FailSafe 2XD buffer (Cambio) following the recommended protocol. The products were run on a 1.2% agarose gel stained with GelRed Nucleic Acid Stain (Biotium) and visualised using a UV transilluminator (BioDoc-it Imaging System).

## Results

### The SVA 5′ to the *FUS* gene is a transcriptional regulator

**Figure 1:**
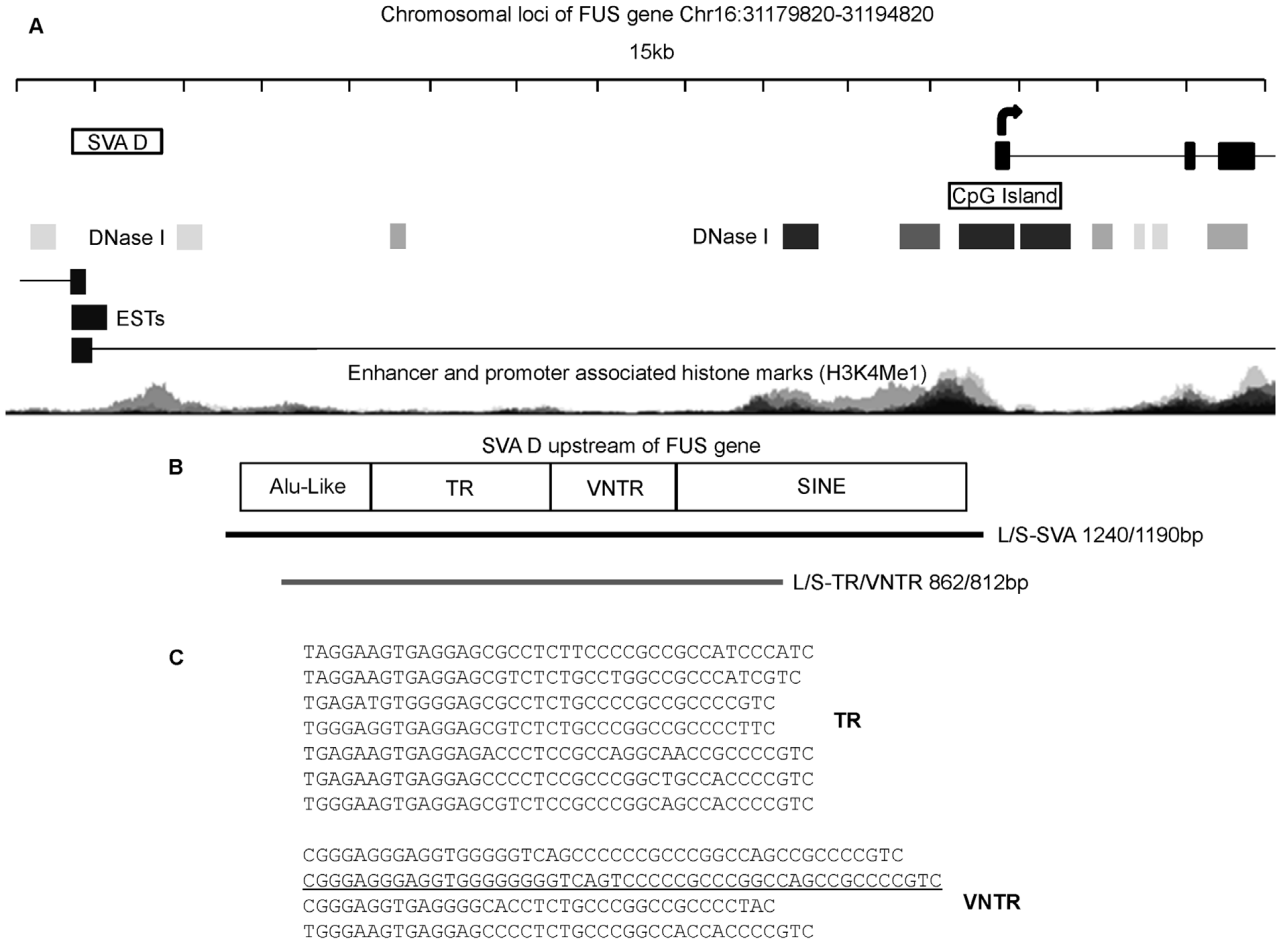
Loci of *FUS* gene and structure of SVA D located upstream. A- Schematic of loci of the *FUS* gene located on chromosome 16. According to UCSC genome browser (Hg18) there are several transcripts with nearly all originating at the transcriptional start site indicated in the diagram. There is a SVA D 9.9kb upstream of this transcriptional start site of the *FUS* gene. This SVA D is present in human and chimpanzees but not other primates. ENCODE data from the genome browser UCSC is summarised indicating the presence of DNase1 clusters, expressed sequence tags (ESTs) and histone modifications associated with enhancers and promoter at this locus. B- Schematic showing the components contributing to the structure of the SVA D located upstream of the *FUS* gene. It contains an Alu-like sequence, a tandem repeat (TR) consisting of 7 copies of a 37–40 bp repeat, a variable number tandem repeat (VNTR) consisting of 3–4 copies of a 37–50 bp repeat and a SINE. This particular SVA is missing the CCCTCT hexamer repeat seen at the 5′ end of a canonical SVA. The fragments cloned into the reporter gene vector (pGL3P) are shown by the black line for the SVA (length 1240/1190 bp) and the grey line for the TR/VNTR (length 862/812 bp). C- Sequence of the 7 copies of the 5′ TR and 3–4 copies of the 3′ VNTR. The repeat underlined is the additional copy found in the long allele which is absent in the short allele of the SVA (sequence in UCSC genome browser corresponds to long allele).

Analysis of the *FUS* gene +/−15 kb identified a large repetitive region approximately 10 kb 5′ of the *FUS* gene and 20 kb from the 5′ end of the PRSS36 gene using the UCSC genome browser (Figure 1A). This repetitive region is part of a larger SVA D element. ENCODE data demonstrated that this SVA overlapped or was adjacent to many features that suggested that it could be regulatory in nature. These included; 1) an area of active histones, H3K4Me1, which are associated with transcription factor binding in genome-wide datasets, 2) human ESTs have been identified which originate and are transcribed in both directions from this location and 3) DNase 1 clusters are located on each side of the SVA (Figure S1). The SVA is present in chimpanzees and humans but not in other primates and does not contain the 5′ CCCTCT hexamer repeat found in a canonical SVA (Figure 1B). Analysis using rVista through the ECR browser identified 146 conserved transcription factor binding sites between the human and chimpanzee SVA sequences; which included a variety of factors such as members of the Sp and GATA families.

The region encompassing this SVA D and the central repetitive region were prepared by PCR from commercially available DNA (Promega), cloned and the sequence validated. On sequence analysis two distinct alleles of the SVA were observed, which differed from one another by one copy of the repeat from the central repetitive region and could therefore be classed as a VNTR. SVAs in general can contain one or two central VNTRs sharing similarities in their sequences but which are distinct from each other. The occurrence of two central VNTRs as opposed to one is seen more frequently in the younger subtypes (D, E, F and F1). The *FUS* SVA appears to belong to the group of SVAs that contain two central repetitive regions as opposed to one. It is in the second of these repetitive regions where the difference between the two alleles is seen. Such variation in only the 2^nd^ domain of the central repeats has been noted in another SVA D located upstream of the *PARK7* gene, which supports gene expression in a reporter gene model *in vitro* [24]. We therefore termed the two repetitive regions in the *FUS* SVA a tandem repeat (TR) and a VNTR when analysed individually and a TR/VNTR when in combination. The two alleles identified were named long (L) and short (S) and the sequence of the TR/VNTR within the SVA is shown in Figure 1C with the additional repeat in the long allele underlined.

**Figure 2:**
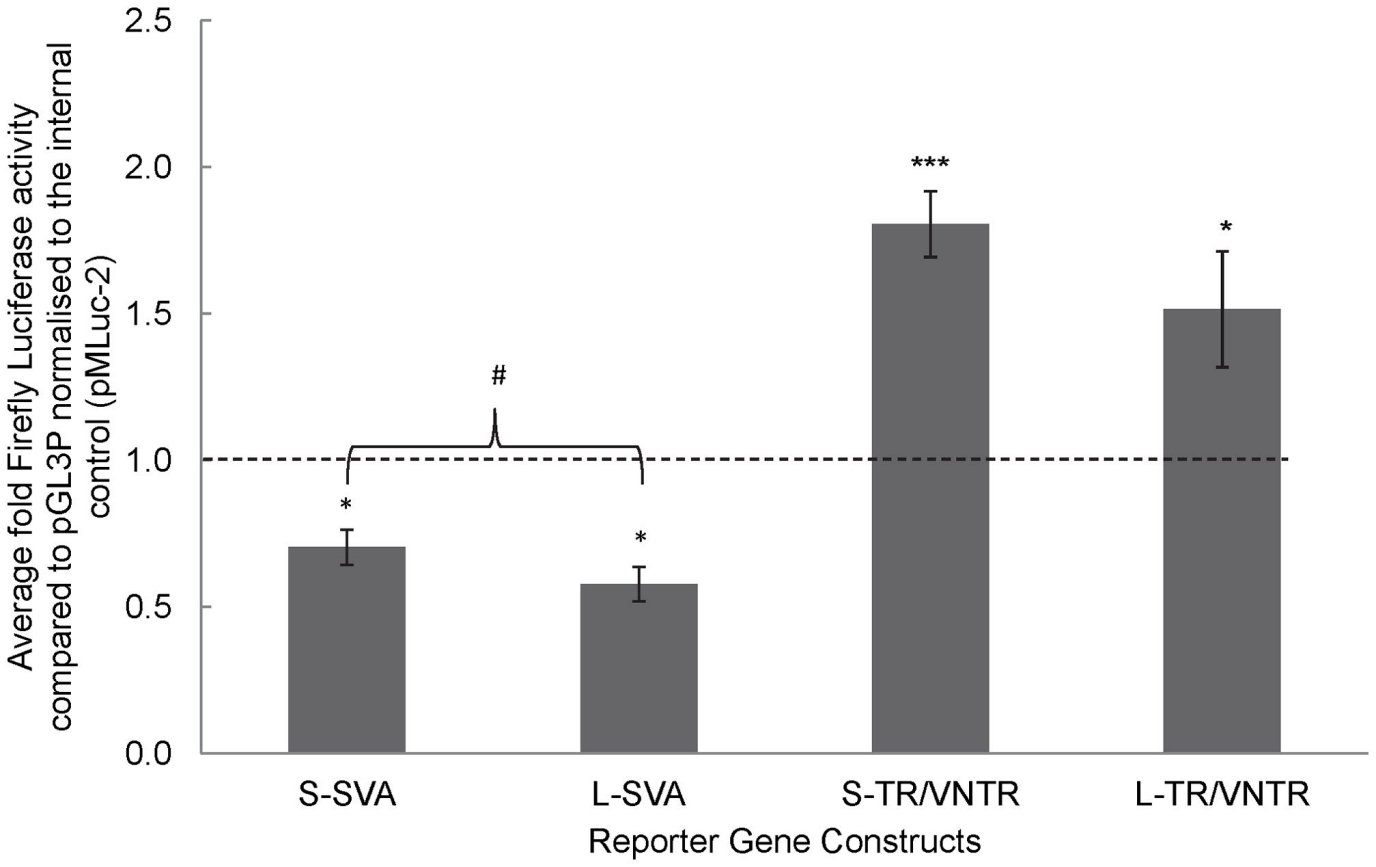
The SVA and the VNTR within show distinct functional properties in a reporter gene construct. Reporter gene constructs containing each allele of the *FUS* SVA and TR/VNTR (long and short) were transfected into the neuroblastoma cell line, SK-N-AS. The fold values of activity demonstrated by each construct compared to pGL3P normalised to the internal control (pMLuc-2) to account for differences in transfection efficiency are displayed. Both alleles when tested as a complete SVA showed repressive function and were significantly different to each other. When the alleles were tested as a smaller fragment consisting of the central TR/VNTR region they both showed enhancer properties. One tailed t-test was used to measure the significance of fold activity of the *FUS* SVA and TR/VNTR over the minimal promoter of the pGL3P vector *P<0.05, ***P<0.001, and to compare the activity of the alleles of the SVA and the TR/VNTR to each other # P<0.05.

Reporter gene constructs were prepared in the pGL3P vector including both variants of the SVA (L-SVA and S-SVA), and the isolated central TR/VNTR (L-TR/VNTR and S-TR/VNTR) (Figure 1B). It was not possible to test the TR and VNTR as separate independent domains as they could not be amplified individually due to their location adjacent to each other, preventing design of a specific primer that would not bind to more than one of the repeats in the *FUS* TR or VNTR. Activity of the constructs was measured in the human neuroblastoma cell line SK-N-AS, which was shown by RT-PCR to express endogenous *FUS*, data not shown. Statistically significant differences were observed in the levels of reporter gene expression supported by the complete SVA or the TR/VNTR compared to the minimal SV40 promoter alone in pGL3P vector (S-SVA p<0.05, L-SVA p<0.05, S-TR/VNTR p<0.001 and L-TR/VNTR p<0.05). Both alleles of the complete SVA repressed reporter gene expression whilst both alleles of the TR/VNTR were activators in this cell line, demonstrating that the SVA may contain multiple and distinct regulatory domains, one of which is a dominant repressor in SK-N-AS cells (Figure 2). When comparing the long and short TR/VNTR constructs no significant difference in the level of reporter gene activity observed was noted, however there was a small but significant difference in the levels of reporter gene expression when these variants were contained within the complete SVA sequence (p<0.05). In both the SVA and TR/VNTR constructs it was the long variant that showed lower activity when compared to the short.

We have previously demonstrated that human specific VNTRs can support tissue specific expression patterns in mouse transgenic models during development [38]. We wanted to address a similar model for the SVA but rather than use a mouse model we used the more convenient and practical chick embryo model [39], [40]. The SVA and TR/VNTR (long allele) domains as used above in the SK-N-AS cell line were inserted into a reporter gene vector we had developed to allow us to visualise activity via hrGFP in the chick embryo model. Briefly the reporter vector phrGFP contained the proximal human *FUS* promoter −160 of the major transcriptional start site to +84 cloned upstream of hrGFP, the TR/VNTR and SVA sequences were inserted immediately upstream of the promoter sequence. The minimal *FUS* promoter does not support gene expression in this model and therefore any marker gene expression is dependent on the cloned regulator.

**Figure 3:**
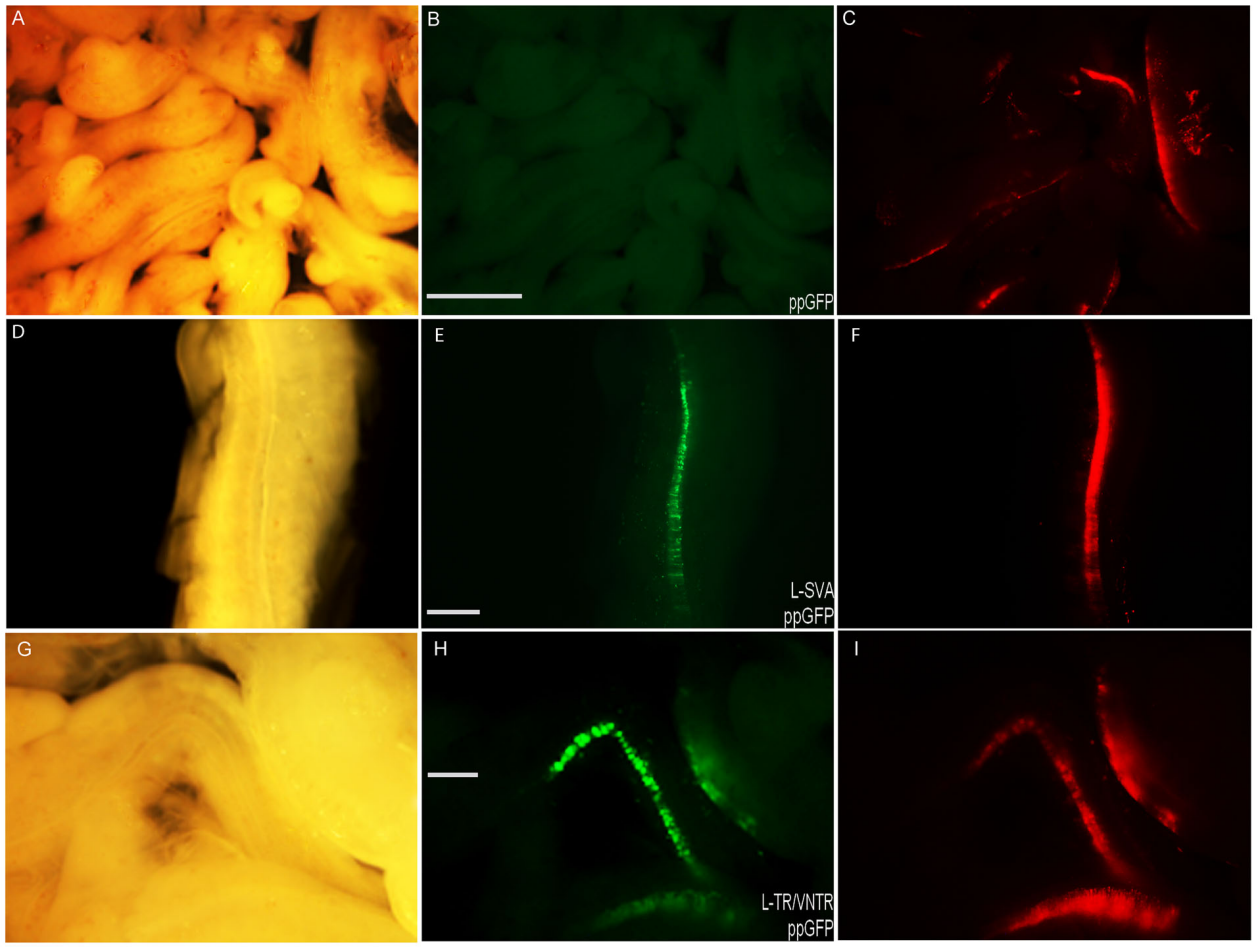
Demonstration of the activity of the L-SVA and L-VNTR presumptive transcriptional regulator in the chick embryo model at stage 22 HH. Chick embryos were electroporated with either a *FUS* proximal promoter GFP (ppGFP) reporter construct (A–C), L-SVA ppGFP-reporter (D–F) or L-TR/VNTR ppGFP-reporter (G–I) at stage 14HH and GFP expression analysed 48 hr later (stage 22HH). Expression could not be detected in the neural tube from the *FUS* proximal promoter sequences alone (B), however when either the L-SVA (E) or L-TR/VNTR (H) sequences were included, GFP reporter gene expression could readily be seen. Panels A, D and G show the corresponding bright field images. Panels C, F and I show the identical fields taken with a red filter to demonstrate the extent of successful electroporation of the neural tube using a control tomato marker expression plasmid. Scale bar in B is 2 mm and in E & H is 1 mm.

The test plasmid was injected into the neural tube and then transfected into cells by electroporation; thus only one side of the neural tube should be transfected. The reporter gene construct was co-injected with an internal control, the tomato reporter plasmid directed by the chick β-actin promoter; the latter acts as an internal control marker for cells which have been successfully transfected. In this manner we addressed the activity and tissue specificity of the L-SVA and the L-TR/VNTR reporter. The series of *FUS* reporter gene constructs were injected into the developing embryo at embryonic stage 14HH and activity analysed at stage 22HH. Endogenous chick *FUS* expression was demonstrated by RT-PCR at this point in the development of the embryo (data not shown). The proximal *FUS* promoter alone did not support sufficient reporter gene expression to be observed in our assay (Figure 3B). However, both the L-SVA and L-TR/VNTR reporter gene constructs supported expression; which was readily observed in the neural tube of the chick embryo (Figure 3E and 3H respectively).

### Genetic variation in the *FUS* SVA

**Figure 4:**
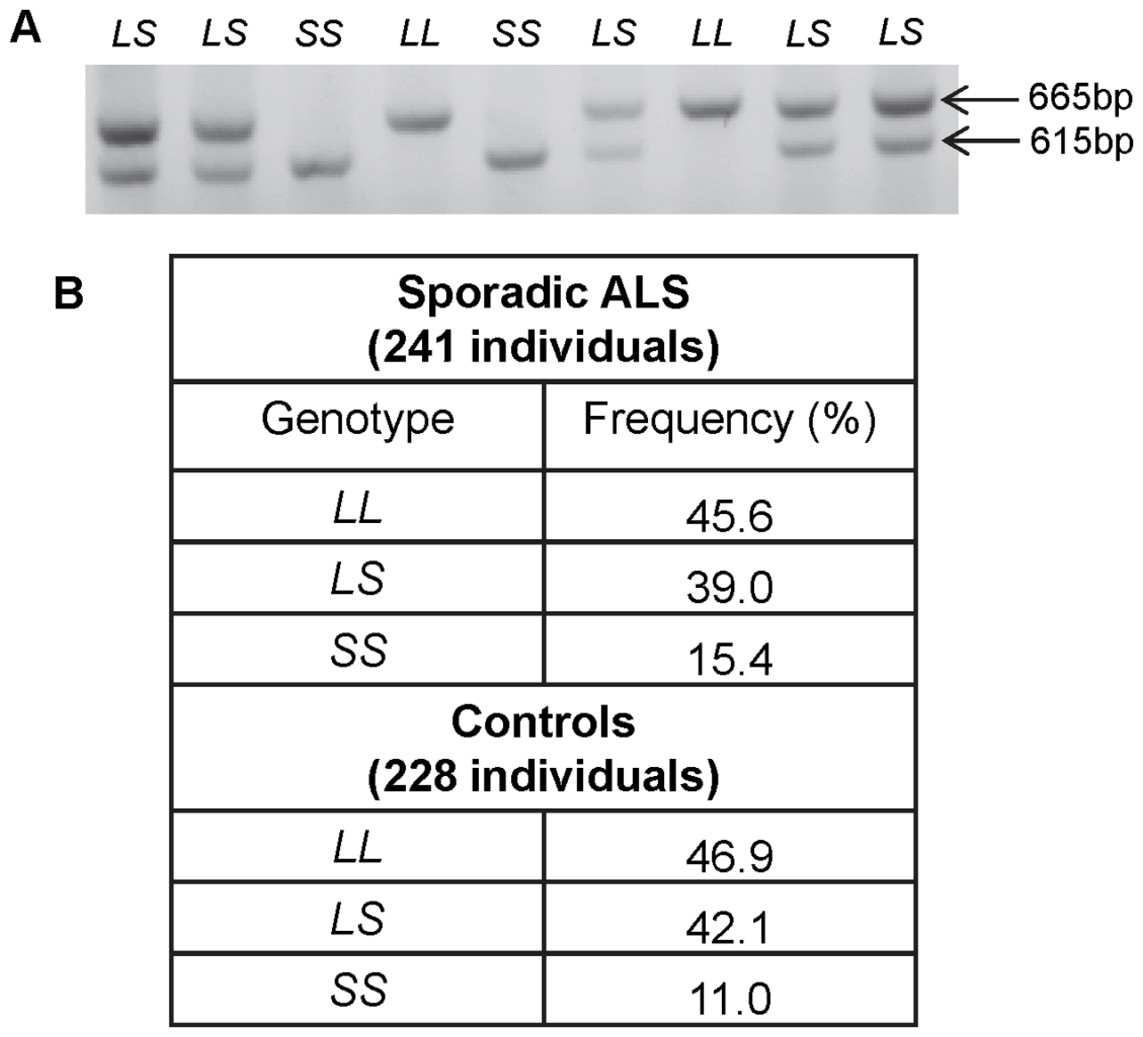
Genotype frequencies of SVA located upstream of the *FUS* gene in a SALS and matched control cohort. A- Example image of the two alleles of the TR/VNTR of the *FUS* SVA run on a 1.2% agarose gel after amplification using PCR. The two alleles identified were named long (*L*) and short (*S*) and the genotype of each individual was determined. One example of each allele was gel extracted and sequenced and corresponded to the previously sequenced and cloned alleles. *L* = 665 bp and *S* = 615 bp. B-. Table showing the percentage of each genotype in the SALS patients (241 individuals) and the matched controls (228 individuals).

It has been previously demonstrated that VNTRs with distinct copy numbers of the repeating element can not only support tissue specific and stimulus inducible reporter gene activity but can also be differentially associated with genetic predisposition to a specific disorder, for example the human transporters for serotonin and dopamine [14], [18], [41], [42]. We therefore expanded the analysis of the polymorphic variation associated with the VNTR within the SVA, addressing this in a cohort of 241 individuals with SALS and 228 matched controls. The genetic variation was analysed by agarose gel electrophoresis of the PCR fragments spanning the TR/VNTR region of the SVA. We found there were only two alleles that could be determined in this cohort (this analysis cannot determine SNP or small insertion/deletion variation within the SVA) (Figure 4A). We confirmed the sequence from both a *L* and *S* allele after gel purification; this demonstrated that the *L* allele corresponded to the sequence found in the UCSC browser for the VNTR of this SVA element (Figure 1C). The two alleles also matched the variants originally identified when cloning the SVA for reporter gene studies from commercially available DNA (Promega). The following genotype frequencies were observed in the SALS cohort 45.6% *LL*, 39% *LS* and 15.4% *SS* and 46.9% *LL*, 42.1% *LS* and 11% *SS* in the matched controls (Figure 4B). Although there was a small difference of 4.4% between the frequency of *SS* individuals in the SALS cohort compared to the matched controls this was found not to be significant when analysed using CLUMP [43]. The T1 2×N table statistic from CLUMP [43] was p = 0.36 and the clumped 2×2 T4 p-value was 0.33, both from 10,000 simulations. CLUMP simulations allow for the small cell values present in sparse 2×N tables such as those in highly multiallelic repeat loci and prevent inflation of the test statistics from generating false positive results.

## Discussion

We have demonstrated that a retrotransposon, of the SVA family, 5′ of the *FUS* gene is both polymorphic and a transcriptional regulator domain. The SVA acted as a classical regulatory domain when analysed in reporter gene constructs *in vitro* and *in vivo*. This data would suggest that the SVA can affect *FUS* gene expression patterns by multiple mechanisms without the requirement for retrotransposition and that distinct polymorphic variants could act to direct differential regulation in response to the same environmental challenges. The transcription factor complement within the cell will be based on a specific stimulus the cell is receiving at any given moment and this synergistic tissue specific and stimulus inducible challenge may result in altering the complement of transcription factors able to direct function from the SVA. There can also be epigenetic variation across SVA elements dependent on the environment for example a change in methylation across retrotransposons was identified in cancer [28].

Both the alleles of the complete SVA and the TR/VNTR domain of the SVA were tested in an *in vitro* reporter gene assay. Distinct from standalone VNTR domains which we have previously addressed, the repetitive region of this particular SVA D contains two adjacent domains comprised of a TR and a VNTR and it was this composite element that was tested in the reporter gene assay. It is interesting that while both the *L* and *S* TR/VNTR regions were enhancers of activity, the intact *L* and *S* SVA acted as a repressor in the SK-N-AS cell line. This suggests that in addition to the activator region in the TR/VNTR the SVA contains a strong active silencer element, flanking this central TR/VNTR region, which is functional in the SK-N-AS neuroblastoma cell line. There are multiple conserved transcription factors within the *FUS* SVA sequence however the action of repressors or enhancers are often determined by the factors available in the cell at any given time therefore further analysis will be required to determine the action of specific transcription factors on the SVA. An alternative explanation for the difference in activity between the TR/VNTR and SVA may be due to proximity of the TR/VNTR domain to the reporter gene when part of the complete SVA element affecting its ability to enhance expression.

There was no significant difference between the activities of the two alleles of the TR/VNTR when tested alone, but there was a significant difference between the two alleles when tested as part of the complete SVA (p<0.05). To further validate the regulatory properties of this domain we tested its properties in the neural tube of the chick embryo. Although *FUS* is a ubiquitously expressed protein this region of the embryo contains motor neurons which are the appropriate cell type to test a domain that might be involved in ALS. As in the cell line model the long allele of the TR/VNTR domain acted as an activator but in this model the long allele of the SVA also demonstrated activator properties which were not exhibited *in vitro*. This would be consistent with our previous analyses of VNTRs from both the serotonin and the dopamine transporters demonstrating cell line specific properties in reporter gene constructs [41], [44], [45] and the intron 2 VNTR from the human serotonin transporter having tissue specific properties in a transgenic mouse model [38]. This particular system of analysing the transcriptional properties of a domain is not quantitative therefore we cannot compare the amount of expression activated by the TR/VNTR and the intact SVA.

Our functional data demonstrated the potential for the *FUS* SVA to act as a transcriptional regulatory element, however only a small difference in the function of the two alleles was observed, although we hypothesise such a difference could be increased upon exposure of the cell to specific challenges. Nevertheless the genotype of the SVA could be a factor which associates with a predisposition to disorders such as ALS. We therefore performed a genotype analysis of the TR/VNTR of the SVA in a SALS and the control cohort from the King's College London MND DNA Bank. This demonstrated two major alleles which we termed *L* and *S* and which correlated to those identified in the cloned commercial DNA (Promega). The frequencies of *LL*, *LS* and *SS* were not found to be significantly different in the sporadic cases compared to the matched controls, although a minor difference could be seen between the frequency of individuals with a SS genotype in SALS and control (15.4% vs 11%), when analysed using CLUMP [43]. This may also reflect in part that *FUS* mutations themselves are rare in SALS (1%) and that we need to address an environmental challenge as a modulator of *FUS* expression. A much larger cohort will be required to validate such variation as an association in the SALS cohort. Our study would not determine the potential SNP or indel variation in the SVA; such variation may be significant for both clinical association with disease and transcriptional properties of the SVA. Precedent for this exists; the long and short alleles of the VNTR within the promoter of the human serotonin transporter gene, in this example there is a genetic association based on GxE interactions, namely a SNP in the long allele makes it clinically similar to the short ‘risk’ allele in genetic associations [46], [47].

In summary we have determined a novel primate tissue specific regulator that could play a role in *FUS* transcriptional regulation. This regulation could be modified by a number of environmental challenges including the changes correlated with the increased RT activity seen in the serum of patients that could affect the epigenetic structure of the *FUS* locus. This regulation could be further modulated by genetic variation in the SVA apart from the VNTR variant observed in this analysis thus allowing for a GxE interaction in any of the diseases' in which *FUS* is implicated.

## Supporting Information

Figure S1 **Locus of** ***FUS*** **gene in the UCSC genome browser.** The *FUS* gene is located on chromosome 16p11.2 and this image is showing 11 kb 5′ of the transcriptional start site of the gene. The region highlighted in the black box corresponds to the SVA D upstream of the *FUS* gene. From the ENCODE data shown in the image there are DNase hypersensitivity clusters, transcription factor binding and enhancer and promoter associated histone marks (H3K4Me1) in the region of this SVA D indicating this is an active region of chromatin.
(TIFF)

## References

[1] Abel O, Powell JF, Andersen PM, Al-Chalabi A (2012) ALSoD: A user-friendly online bioinformatics tool for amyotrophic lateral sclerosis genetics. Hum Mutat 33: 1345–1351. 22753137 10.1002/humu.22157

[2] Chio A, Calvo A, Moglia C, Ossola I, Brunetti M, et al.. (2011) A de novo missense mutation of the FUS gene in a “true” sporadic ALS case. Neurobiol Aging 32: 553 e523–556. 10.1016/j.neurobiolaging.2010.05.016 PMC2972379 20598774

[3] Lai SL, Abramzon Y, Schymick JC, Stephan DA, Dunckley T, et al.. (2011) FUS mutations in sporadic amyotrophic lateral sclerosis. Neurobiol Aging 32: 550 e551–554. 10.1016/j.neurobiolaging.2009.12.020 PMC2891336 20138404

[4] Sproviero W, La Bella V, Mazzei R, Valentino P, Rodolico C, et al.. (2012) FUS mutations in sporadic amyotrophic lateral sclerosis: clinical and genetic analysis. Neurobiol Aging 33: 837 e831–835. 10.1016/j.neurobiolaging.2011.10.005 22055719

[5] Corrado L, Del Bo R, Castellotti B, Ratti A, Cereda C, et al (2010) Mutations of FUS gene in sporadic amyotrophic lateral sclerosis. J Med Genet 47: 190–194. 19861302 10.1136/jmg.2009.071027

[6] Vance C, Rogelj B, Hortobagyi T, De Vos KJ, Nishimura AL, et al (2009) Mutations in FUS, an RNA processing protein, cause familial amyotrophic lateral sclerosis type 6. Science 323: 1208–1211. 19251628 10.1126/science.1165942 PMC4516382

[7] Kwiatkowski TJ Jr, Bosco DA, Leclerc AL, Tamrazian E, Vanderburg CR, et al (2009) Mutations in the FUS/TLS gene on chromosome 16 cause familial amyotrophic lateral sclerosis. Science 323: 1205–1208. 19251627 10.1126/science.1166066

[8] Deng HX, Zhai H, Bigio EH, Yan J, Fecto F, et al (2010) FUS-immunoreactive inclusions are a common feature in sporadic and non-SOD1 familial amyotrophic lateral sclerosis. Ann Neurol 67: 739–748. 20517935 10.1002/ana.22051 PMC4376270

[9] Mitchell JC, McGoldrick P, Vance C, Hortobagyi T, Sreedharan J, et al (2013) Overexpression of human wild-type FUS causes progressive motor neuron degeneration in an age- and dose-dependent fashion. Acta Neuropathol 125: 273–288. 22961620 10.1007/s00401-012-1043-z PMC3549237

[10] Davidson S, Lear M, Shanley L, Hing B, Baizan-Edge A, et al (2011) Differential activity by polymorphic variants of a remote enhancer that supports galanin expression in the hypothalamus and amygdala: implications for obesity, depression and alcoholism. Neuropsychopharmacology 36: 2211–2221. 21716262 10.1038/npp.2011.93 PMC3176579

[11] Paredes UM, Bubb VJ, Haddley K, Macho GA, Quinn JP (2011) An evolutionary conserved region (ECR) in the human dopamine receptor D4 gene supports reporter gene expression in primary cultures derived from the rat cortex. BMC Neurosci 12: 46. 21599953 10.1186/1471-2202-12-46 PMC3121617

[12] Shanley L, Davidson S, Lear M, Thotakura AK, McEwan IJ, et al (2010) Long-range regulatory synergy is required to allow control of the TAC1 locus by MEK/ERK signalling in sensory neurones. Neurosignals 18: 173–185. 21160161 10.1159/000322010 PMC3718575

[13] Vasiliou SA, Ali FR, Haddley K, Cardoso MC, Bubb VJ, et al (2012) The SLC6A4 VNTR genotype determines transcription factor binding and epigenetic variation of this gene in response to cocaine in vitro. Addict Biol 17: 156–170. 21309950 10.1111/j.1369-1600.2010.00288.x

[14] Haddley K, Bubb VJ, Breen G, Parades-Esquivel UM, Quinn JP (2012) Behavioural Genetics of the Serotonin Transporter. Curr Top Behav Neurosci. 10.1007/7854_2011_186 22261701

[15] Ali FR, Vasiliou SA, Haddley K, Paredes UM, Roberts JC, et al (2010) Combinatorial interaction between two human serotonin transporter gene variable number tandem repeats and their regulation by CTCF. J Neurochem 112: 296–306. 19860858 10.1111/j.1471-4159.2009.06453.x PMC2848977

[16] Miyajima F, Quinn JP, Horan M, Pickles A, Ollier WE, et al (2008) Additive effect of BDNF and REST polymorphisms is associated with improved general cognitive ability. Genes Brain Behav 7: 714–719. 18518926 10.1111/j.1601-183X.2008.00409.x

[17] Roberts J, Scott AC, Howard MR, Breen G, Bubb VJ, et al (2007) Differential regulation of the serotonin transporter gene by lithium is mediated by transcription factors, CCCTC binding protein and Y-box binding protein 1, through the polymorphic intron 2 variable number tandem repeat. J Neurosci 27: 2793–2801. 17360901 10.1523/JNEUROSCI.0892-06.2007 PMC6672559

[18] Guindalini C, Howard M, Haddley K, Laranjeira R, Collier D, et al (2006) A dopamine transporter gene functional variant associated with cocaine abuse in a Brazilian sample. Proc Natl Acad Sci U S A 103: 4552–4557. 16537431 10.1073/pnas.0504789103 PMC1450209

[19] Klenova E, Scott AC, Roberts J, Shamsuddin S, Lovejoy EA, et al (2004) YB-1 and CTCF differentially regulate the 5-HTT polymorphic intron 2 enhancer which predisposes to a variety of neurological disorders. J Neurosci 24: 5966–5973. 15229244 10.1523/JNEUROSCI.1150-04.2004 PMC6729234

[20] MacKenzie A, Quinn JP (2004) Post-genomic approaches to exploring neuropeptide gene mis-expression in disease. Neuropeptides 38: 1–15. 15003710 10.1016/j.npep.2003.09.004

[21] Hing B, Davidson S, Lear M, Breen G, Quinn J, et al (2012) A Polymorphism Associated with Depressive Disorders Differentially Regulates Brain Derived Neurotrophic Factor Promoter IV Activity. Biol Psychiatry 71: 618–626. 22265241 10.1016/j.biopsych.2011.11.030 PMC3712170

[22] Doolittle WF (2013) Is junk DNA bunk? A critique of ENCODE. Proc Natl Acad Sci U S A. 10.1073/pnas.1221376110 PMC3619371 23479647

[23] Kavanagh DH, Dwyer S, O'Donovan MC, Owen MJ (2013) The ENCODE project: implications for psychiatric genetics. Mol Psychiatry. 10.1038/mp.2013.13 23478746

[24] Savage AL, Bubb VJ, Breen G, Quinn JP (2013) Characterisation of the potential function of SVA retrotransposons to modulate gene expression patterns. BMC Evol Biol 13: 101. 23692647 10.1186/1471-2148-13-101 PMC3667099

[25] Hancks DC, Kazazian HH Jr (2012) Active human retrotransposons: variation and disease. Curr Opin Genet Dev 22: 191–203. 22406018 10.1016/j.gde.2012.02.006 PMC3376660

[26] Baillie JK, Barnett MW, Upton KR, Gerhardt DJ, Richmond TA, et al (2011) Somatic retrotransposition alters the genetic landscape of the human brain. Nature 479: 534–537. 22037309 10.1038/nature10531 PMC3224101

[27] Faulkner GJ (2011) Retrotransposons: mobile and mutagenic from conception to death. FEBS Lett 585: 1589–1594. 21477589 10.1016/j.febslet.2011.03.061

[28] Szpakowski S, Sun X, Lage JM, Dyer A, Rubinstein J, et al (2009) Loss of epigenetic silencing in tumors preferentially affects primate-specific retroelements. Gene 448: 151–167. 19699787 10.1016/j.gene.2009.08.006 PMC2783545

[29] Wang H, Xing J, Grover D, Hedges DJ, Han K, et al (2005) SVA elements: a hominid-specific retroposon family. J Mol Biol 354: 994–1007. 16288912 10.1016/j.jmb.2005.09.085

[30] Bantysh OB, Buzdin AA (2009) Novel family of human transposable elements formed due to fusion of the first exon of gene MAST2 with retrotransposon SVA. Biochemistry (Mosc) 74: 1393–1399. 19961423 10.1134/s0006297909120153

[31] Hancks DC, Ewing AD, Chen JE, Tokunaga K, Kazazian HH Jr (2009) Exon-trapping mediated by the human retrotransposon SVA. Genome Res 19: 1983–1991. 19635844 10.1101/gr.093153.109 PMC2775590

[32] Damert A, Raiz J, Horn AV, Lower J, Wang H, et al (2009) 5′-Transducing SVA retrotransposon groups spread efficiently throughout the human genome. Genome Res 19: 1992–2008. 19652014 10.1101/gr.093435.109 PMC2775593

[33] Alfahad T, Nath A (2013) Retroviruses and amyotrophic lateral sclerosis. Antiviral Res 99: 180–187. 23707220 10.1016/j.antiviral.2013.05.006 PMC3723705

[34] Steele AJ, Al-Chalabi A, Ferrante K, Cudkowicz ME, Brown RH Jr, et al (2005) Detection of serum reverse transcriptase activity in patients with ALS and unaffected blood relatives. Neurology 64: 454–458. 15699374 10.1212/01.WNL.0000150899.76130.71

[35] Andrews WD, Tuke PW, Al-Chalabi A, Gaudin P, Ijaz S, et al (2000) Detection of reverse transcriptase activity in the serum of patients with motor neurone disease. J Med Virol 61: 527–532. 10897073 10.1002/1096-9071(200008)61:4<527::aid-jmv17>3.0.co;2-a

[36] Douville R, Liu J, Rothstein J, Nath A (2011) Identification of active loci of a human endogenous retrovirus in neurons of patients with amyotrophic lateral sclerosis. Ann Neurol 69: 141–151. 21280084 10.1002/ana.22149 PMC3052883

[37] Hamburger V, Hamilton HL (1951) A series of normal stages in the development of the chick embryo. Journal of Morphology 88: 49–92. 24539719

[38] MacKenzie A, Quinn J (1999) A serotonin transporter gene intron 2 polymorphic region, correlated with affective disorders, has allele-dependent differential enhancer- like properties in the mouse embryo. Proc Natl Acad Sci U S A 96: 15251–15255. 10611371 10.1073/pnas.96.26.15251 PMC24806

[39] Uchikawa M (2008) Enhancer analysis by chicken embryo electroporation with aid of genome comparison. Dev Growth Differ 50: 467–474. 18422684 10.1111/j.1440-169X.2008.01028.x

[40] Uchikawa M, Ishida Y, Takemoto T, Kamachi Y, Kondoh H (2003) Functional analysis of chicken Sox2 enhancers highlights an array of diverse regulatory elements that are conserved in mammals. Dev Cell 4: 509–519. 12689590 10.1016/s1534-5807(03)00088-1

[41] Haddley K, Vasiliou AS, Ali FR, Paredes UM, Bubb VJ, et al (2008) Molecular genetics of monoamine transporters: relevance to brain disorders. Neurochem Res 33: 652–667. 17960477 10.1007/s11064-007-9521-8

[42] Brotons O, O'Daly OG, Guindalini C, Howard M, Bubb J, et al (2011) Modulation of orbitofrontal response to amphetamine by a functional variant of DAT1 and in vitro confirmation. Mol Psychiatry 16: 124–126. 20856247 10.1038/mp.2009.6

[43] Sham PC, Curtis D (1995) Monte Carlo tests for associations between disease and alleles at highly polymorphic loci. Ann Hum Genet 59: 97–105. 7762987 10.1111/j.1469-1809.1995.tb01608.x

[44] Paredes UM, Quinn JP, D'Souza UM (2012) Allele-specific transcriptional activity of the variable number of tandem repeats in 5′ region of the DRD4 gene is stimulus specific in human neuronal cells. Genes Brain Behav. 10.1111/j.1601-183X.2012.00857.x 23013251

[45] Michelhaugh SK, Fiskerstrand C, Lovejoy E, Bannon MJ, Quinn JP (2001) The dopamine transporter gene (SLC6A3) variable number of tandem repeats domain enhances transcription in dopamine neurons. J Neurochem 79: 1033–1038. 11739616 10.1046/j.1471-4159.2001.00647.x

[46] Wray NR, James MR, Gordon SD, Dumenil T, Ryan L, et al (2009) Accurate, Large-Scale Genotyping of 5HTTLPR and Flanking Single Nucleotide Polymorphisms in an Association Study of Depression, Anxiety, and Personality Measures. Biol Psychiatry 66: 468–476. 19541292 10.1016/j.biopsych.2009.04.030 PMC3060567

[47] Wendland JR, Martin BJ, Kruse MR, Lesch KP, Murphy DL (2006) Simultaneous genotyping of four functional loci of human SLC6A4, with a reappraisal of 5-HTTLPR and rs25531. Mol Psychiatry 11: 224–226. 16402131 10.1038/sj.mp.4001789

