# Correction: An Evaluation of a SVA Retrotransposon in the *FUS* Promoter as a Transcriptional Regulator and Its Association to ALS

**DOI:** 10.1371/journal.pone.0101398

**Published:** 2014-06-20

**Authors:** The PLOS ONE Staff

There is an error in the Author Contributions. The third author, Kejhal Khursheed, should be noted as contributing equally to this work along with the first and second authors, Abigail L. Savage and Thomas P. Wilm. The publisher apologizes for this error.
