# Supplementary figures and images for: An Evaluation of a SVA Retrotransposon in the FUS Promoter as a Transcriptional Regulator and Its Association to ALS
Source: PLoS One. 2014 Mar 7;9(3):e90833. doi: 10.1371/journal.pone.0090833 (PMC3946630; doi:10.1371/journal.pone.0090833)

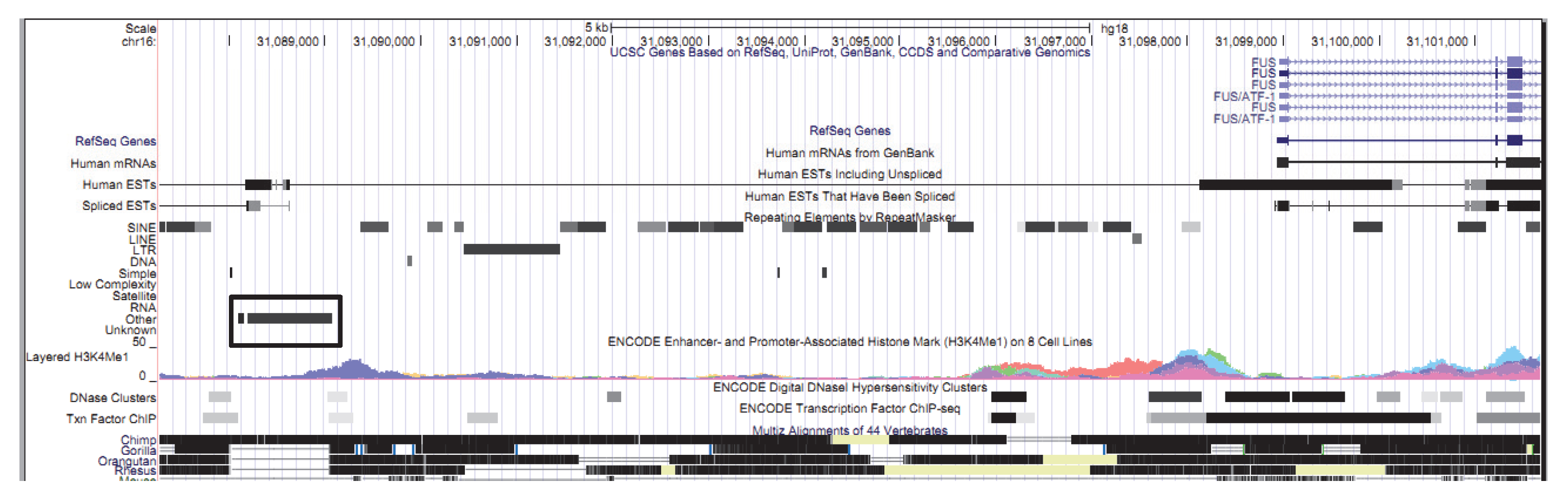

Supplement: Figure S1 — Locus of FUS gene in the UCSC genome browser. The FUS gene is located on chromosome 16p11.2 and this image is showing 11 kb 5′ of the transcriptional start site of the gene. The region highlighted in the black box corresponds to the SVA D upstream of the FUS gene. From the ENCODE data shown in the image there are DNase hypersensitivity clusters, transcription factor binding and enhancer and promoter associated histone marks (H3K4Me1) in the region of this SVA D indicating this is an active region of chromatin. (TIFF) [file pone.0090833.s001.tiff]
